# Stevens–Johnson syndrome associated with daclatasvir and sofosbuvir co-therapy in hepatitis C treatment: a rare case report

**DOI:** 10.1097/MS9.0000000000003743

**Published:** 2025-08-28

**Authors:** Waseem Sajjad, Ubaid Ur Rehman, Areeba Inam, Iftikhar Alam, Shahmir Ghafoor, Hussain Danyal Razzaq, Taqweem Khwajakhail, Syed Hasssan Mohi Ud Din Gillani, Hassan Razzaq, Ihsan Qamar, Rahmat Ali, Zabeeh Ullah

**Affiliations:** a King Edward Medical University, Mayo Hospital, Lahore, Pakistan; b Ayub Medical College, Abbotabad, Pakistan; c Punjab Medical College, Faisalabad, Pakistan; d Islam Medical College, Sialkot, Pakistan; e Mayo Hospital, Lahore, Pakistan; f Spinghar University, Kabul, Afghanistan; g Landmark Medical Center, Rhode Island, United States

**Keywords:** antiviral drugs, daclatasvir, hepatitis C infection, sofosbuvir, Stevens–Johnson syndrome

## Abstract

**Background:**

Stevens–Johnson syndrome (SJS) is an uncommon, severe skin and mucous membrane illness that is frequently triggered by some medications and infections like herpes simplex virus infection or *Mycoplasma pneumoniae* infection.

**Case presentation:**

This case report describes an uncommon but severe side effect that happened after taking two antiviral medications, sofosbuvir and daclatasvir, to treat hepatitis C virus infection. A 54-year-old man developed mild rash after taking prescribed medications for 3 days, which rapidly progressed to typical SJS with significant mucosal and skin involvement. With passing days, the patient’s condition kept on getting worse despite conservative care and cessation of sofosbuvir and daclatasvir. The patient was given continuous supportive and symptomatic treatment, but despite of intensive care, the patient expired on fourth day of hospital admission.

**Discussion:**

“Daclatasvir and Sofosbuvir Co-therapy” is considered an effective regime for Hepatitis C (Hep C) infection; however, we have a rare occurrence of SJS due to this drug regime that has not been reported in literature previously. By presenting this case, we hope to offer valuable insights that will help clinicians in the identification and management of similar cases ensuring patient safety while maintaining the benefits of this effective regime.

**Conclusion:**

Even though SJS is rare, the possibility that it might have occurred due to sofosbuvir–daclatasvir highlights the need for clinicians to be vigilant while prescribing this regime and educate the patients about the signs and symptoms of SJS. Clinical practices can lower hazards and improve patient safety by implementing these measures.

## Introduction

Stevens–Johnson syndrome (SJS) is an uncommon, severe skin and mucous membrane illness that is frequently triggered by some medications, infections, and autoimmune reactions, and in some cases, it can be idiopathic. Usually, it starts with flu-like symptoms, then progresses to a painful rash that blisters and spreads, and after a few days, the top layer of the affected skin dies, sheds, and starts to recover^[1]^.Antipsychotics, anti-seizure drugs, anti-gout drugs, painkillers like paracetamol and ibuprofen, and antibiotics like penicillin are among the drugs that have been linked to SJS. Herpes Simplex virus and *Mycoplasma pneumoniae* are among the infections that have been linked to SJS. Roughly 1–7 occurrences of SJS per million individuals occur annually^[2,3]^. According to a study in the USA, the incidence rate is 1.58–2.26 cases/million people, but the overall incidence of SJS is unclear till now^[4]^. With any of its related causes, the risk of SJS is still minimal. Previous studies have reported mortality rates for SJS to be approximately 19%–29%^[5]^. The dangers associated with declining immune function appear to be correlated with the increased incidence of the condition and the corresponding mortality rate in immunocompromised patients^[5]^. Hepatitis C (Hep C) is treated with the antiviral drug combination of sofosbuvir and daclatasvir. If the patient develops cirrhosis, other drugs such as interferon and ribavirin may be given in combination. Sofosbuvir and daclatasvir are directly acting antiviral medications that block the HCV non-structural proteins (NS5A5) that are essential for its replication and survival^[6]^.

**HIGHLIGHTS**

- A rare case of Stevens–Johnson syndrome (SJS) linked to daclatasvir and sofosbuvir co-therapy in hepatitis C treatment.
- Highlights the need for early recognition of severe cutaneous adverse reactions in antiviral therapy.
- Emphasizes the importance of pharmacovigilance in direct-acting antiviral regimens of these drugs.
- Discusses the clinical presentation, diagnosis, and management of SJS.
- Raises awareness about potential life-threatening drug reactions in hepatitis C patients when treated with these drugs.

Due to the lack of evidence in the literature, this case report is significant to report regarding the relationship between sofosbuvir–daclatasvir and SJS. Investigating this possible association further is critical since SJS, although uncommon, is a serious and potentially fatal illness, and sofosbuvir–daclatasvir is a drug combination that is frequently used for HCV.

Considering drug-induced SJS, sofosbuvir and daclatasvir were withdrawn, and the patient was given continuous care, but despite of extensive care, the patient expired on fourth day of hospital admission. The benefits of both drugs to humans cannot be ignored as it is considered to be the safest drug combination in modern medicine in HCV treatment plans. The aim of reporting this case to make the physician cautious and keeping this possible side of SJS in mind while prescribing this drug regime and educate the patients to be vigilant and promptly consult if they develop any rash. This work has been reported according to “SCARE” and “TITAN” guidelines^[7,8]^.

## Case report

**Figure 1.**
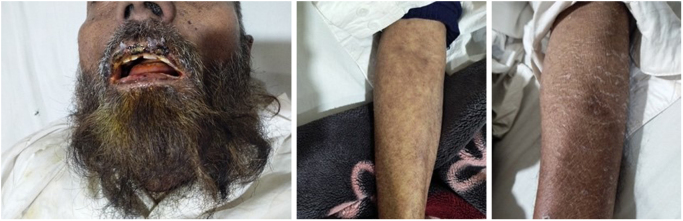
Pictures of patient with mucosal ulceration and skin changes of different parts of the body showing typical illustration of SJS.

A 54-year-old male, with no previous history of any allergy to food, drug, or any other substance, presented to the emergency department with an altered state of consciousness and dyspnea for the last 2 hours. Examination revealed that the patient was in severe dehydration and generalized weakness with a high-grade fever (103°F), swollen eyes with discharge, ulceration and sloughing of the oral mucosa with hemorrhage, and crustings involving buccal mucosa and mucocutaneous tissues of the lips and perioral areas (Fig. 1), as well as other areas of the body (Fig. 1).

Vermillion border and ulcerated perioral lesions were covered with grayish-white topically applied ointment. There was cervical lymphadenopathy. Ocular findings included periorbital oedema, conjunctival hyperemia, and copious mucopurulent discharge from the eyes. Eyes were bright red, with prominent blood vessels and very little visible white scleral tissue. Drooling and dribbling were evident due to excess salivation. His weight was 67 kg, with an average body build.

The attendant explained that the patient was diagnosed with Hep C a week ago. He was prescribed the antiviral drugs “sofosbuvir” and “daclatasvir.” The dosage of the drugs included Tablet Sofos (sofosbuvir) 400 mg OD and Tablet DACLA (daclatasvir) 60 mg OD. According to him, the patient was showing complete compliance with the medication dosage and timing. After taking the medication for 3 days, the patient started developing a mild skin rash that transformed into small bullous lesions all over the body, which progressively worsened with passing days. The patient developed a mild fever. The patients’ symptoms were treated conservatively at home with over-the-counter available antipyretics, analgesics, and ointments, but no improvement was seen.

The patient kept on continuing the prescribed drugs for hepatitis, and his conditions kept on worsening until he developed the current condition 2 hours ago and was received in the Emergency Department. No other medications, herbal supplements, or recent vaccinations were reported by the patient’s attendant. The patient had no past history of similar reactions. There was no recent history of recent infection. The temporal relationship of taking sofosbuvir–daclatasvir and development of rash raised suspicion for a drug-induced etiology. Thus, erythema multiforme or SJS was probable diagnosis on history.

On complete evaluation, the patient has hot, dried hemorrhagic crusts all over his body. He was evaluated for state of consciousness, and a score of 9 out of 15 (E3V2M4) was established on the Glasgow Coma Scale. Pupils were round and reactive to light. Planter reflexes were reduced. Generalized abdominal tenderness was present, which was more severe in the left hypochondrium and left lumber region. Heart sounds were normal but feeble. Blood pressure was 90/65 mm Hg. Arterial blood gas analysis showed a combination of metabolic acidosis and compensatory respiratory alkalosis. The hypoxemia (low pO2 and %sO2c) indicated impaired oxygenation. The elevated alveolar–arterial oxygen gradient further supported a significant gas exchange abnormality, potentially indicating a ventilation–perfusion mismatch or impaired diffusion. The SCORTEN (SCORe of Toxic Epidermal Necrosis) assessment score was 2 on presentation with age more than 40 years and serum urea nitrogen levels more than 28 mg/dl. The SCORTEN assessment score is a validated clinical severity-of-illness score used to predict mortality in patients with SJS. The predicted mortality associated with a SCORTEN score of 2 is approximately 12.1%. Detailed lab investigations of the patient are given in Tables 1–3 on day 1, i.e., admission day, day 2, and day 3, respectively. Renal function tests showed markedly increased urea and creatinine. Serum electrolytes showed decreased sodium and normal chloride and potassium levels. Note that only selective and necessary investigations are done on follow-up days owing to limited resources and patient overload. On the day 3 of admission, liver function test showed increased aspartate aminotransferase (AST), Alanine aminotransferase (ALT), Alkaline phosphatase (ALP), and total bilirubin. Renal function tests showed markedly increased urea and creatinine demonstrating worsening renal function.

**Table 1:** **Lab investigations** **of the patient on admission day, i.e., day 1**

Test	Reference	Unit	Results
Complete blood county			
WBC	4–11	10 × 9/L	2.3
RBC	3.5–5.5	10 × 12/L	3.46
HGB	13–16	g/dl	9.4
HCT	36–46	%	28.2
MCV	76–96	fL	81.5
MCHC	31.5–34.5	g/dl	33.5
PLT	150–450	10 × 9/L	49
Neutrophils	40–80	%	4.9
Lymphocytes	20–40	%	85.9
Monocytes	2–8	%	9.2
Eosinophil	1–5	%	0
MCH	27–31	pg	27.3
Liver function tests			
AST	10–40	IU/L	102
ALT	10–40	IU/L	48
ALP	44–147	IU/L	9
Total bilirubin	0.1–1.20	mg/dl	1.24
Renal function tests			
Serum Urea	10–50	mg/dl	190
Serum Creatinine	0.7–1.2	mg/dl	3.41
Serum electrolytes (Na, Cl, and K) and ions (Ca and P)			
Sodium	134–146	mE/L	153
Chloride	96–106	mE/L	73
Potassium	3.5–5.2	mE/L	14.8
Calcium	8.4–10.6	mg/dl	8.6
Phosphorus	3.0–4.50	mg/dl	4.8
Cardiac enzymes			
LDH	140–280	IU/L	193
CPK	26–190	IU/L	27
CK-MB	5–25	IU/L	30

ALP, alkaline phosphatase; ALT, alanine aminotransferase; AST, aspartate aminotransferase.

**Table 2:** Selective lab investigations repeated on day 2 of admission of patient

Test	Reference	Unit	Results
Renal Function Tests			
Serum urea	10–50	mg/dl	251
Serum creatinine	0.7–1.2	mg/dl	3.57
Serum electrolytes (sample type – arterial)			
Sodium	134–146	mE/L	128
Chloride	96–106	mE/L	94.9
Potassium	3.5–5.2	mE/L	4.08

**Table 3:** Selective lab investigations repeated on day 3 of admission of patient

Test	Reference	Unit	Results
Liver function tests			
AST	10–40	IU/L	289
ALT	10–40	IU/L	399
ALP	44–147	IU/L	452
Total bilirubin	0.1–1.20	mg/dl	4.7
Renal function tests			
Serum urea	10–50	mg/dl	265
Serum creatinine	0.7–1.2	mg/dl	3.98
Serum electrolytes (Na, K, and Cl)			
Sodium	134–146	mE/L	132
Chloride	96–106	mE/L	97
Potassium	3.5–5.2	mE/L	3.5

ALP, alkaline phosphatase; ALT, alanine aminotransferase; AST, aspartate aminotransferase.

Sofosbuvir and daclatasvir were withdrawn, and the patient was treated conservatively. The patient was given extreme supportive and symptomatic treatment but the response was not satisfactory despite intensive care and the patient expired on the fourth day of hospital admission.

Informed consent was taken by the attendant.

## Discussion

Depending on the level of involvement, related consequences, and mortality, drug rashes can be classified as mild, moderate, severe, or life-threatening. SJS, toxic epidermal necrolysis, erythema multiforme, acute generalized exanthematous pustulosis, and any rash requiring systemic corticosteroid therapy are among the reactions that might be fatal^[9]^. Treatment for mild and moderate cases can be improved with topical drugs and supportive care, but permanent cessation of therapy is also necessary for severe and life-threatening cases.

Sofosbuvir–daclatasvir have a strong and well-established safety profile, with minimal side effects reported in most large-scale trials and case reports. With sofosbuvir and other medications like ribavirin and ledipasvir, rash incidence has been documented. Limited research has been done on the incidence of rash caused only by sofosbuvir^[10]^. Serious skin reactions like SJS are extremely rare and have not been widely documented with this drug combination. Since, as far as we are aware, there has never been a single report of SJS linked to the administration of the drug combination of sofosbuvir and daclatasvir, our case is significant to report. It is quite possible that our patient’s continued usage of sofosbuvir and daclatasvir following the onset of skin eruptions contributed to the development of SJS.

Erythema multiforme major, also known as SJS, is a potentially fatal inflammatory mucocutaneous drug reaction that falls between erythema multiforme minor and toxic epidermal necrolysis. The former is typified by targetoid cutaneous lesions that cover less than 10% of the body surface area, while the latter is characterized by widespread mucocutaneous involvement that affects 30–100% of the skin surface. Uncertain upper respiratory tract symptoms that last up to 2 weeks are a typical first sign of SJS^[11]^.

Patients may experience chills, headaches, fever, sore throats, and malaise throughout this time. Studies have shown that prolonged fever may occur in up to 85% of instances, even in the absence of an accompanying illness. Nevertheless, a fever that lasts longer than 4 weeks may raise suspicions of a concurrent infection. Mucocutaneous lesions then appear quickly after this. Mucous membrane erosions that cause pain are frequent and can occur in any part of the body during an illness, including the lip, mouth, conjunctiva, nasal cavity, urethra, vagina, gastrointestinal tract, and respiratory tract. About 90% of afflicted patients have visible mucous membrane involvement. Roughly 1–7 occurrences of SJS per million individuals occur annually^[12]^.

With any of its related causes, the risk of SJS is still minimal. Between 5% and 15% of cases of SJS result in death. The dangers associated with declining immune function appear to be correlated with the increased incidence of the condition and the corresponding mortality rate in immunocompromised patients^[2]^. With >10% skin surface desquamation and mucosal surface involvement, our patient experienced distinctive and fast-increasing painful, hot, and dry hemorrhagic crusts across his body, which were consistent with SJS. Since mucosal involvement was observed and there was no sudden onset within hours, bullous drug eruptions were excluded. Exfoliative erythroderma, which displays erythema, scaling, and induration without blisters or mucosal involvement, was ruled out as our patient had mucosal involvement. Toxic epidermal necrolysis, which often affects more than 30% of the skin’s surface, were first ruled out as in our patient more than 10% and less than 30% percent body surface was involved. Given the severe skin sloughing and discomfort our patient was experiencing, viral exanthems were not probable. Since there was no fever, toxic characteristics, eosinophilia, or lymphadenopathy, staphylococcal scalded skin syndrome, medication response with eosinophilia, and systemic symptoms were ruled out^[13]^.

Although the exact mechanism of development of SJS by sofosbuvir and daclatasvir is unknown, plausible explanations include immune-mediated damage in a genetically susceptible individual, Fas-mediated apoptosis, or cytotoxic T-cell-mediated lysis of keratinocytes. Further research is required to identify the precise mechanisms of SJS associated with sofosbuvir–daclatasvir drug combination, which is considered the safest in the contemporary medicine^[14]^.

Keeping in mind the severity of the consequences of the SJS, stopping the offending medication is essential for the management of SJS in this condition, in addition to fluids, nourishment, antibiotics, and supportive care. Oral corticosteroids or intravenous immunoglobulins may be used in some situations^[15]^. However, despite the discontinuation of sofosbuvir–daclatasvir, and intensive care with supportive and symptomatic treatment including topical emollients and steroids, our patient expired on fourth day of hospital admission.

## Limitations

This case report has several significant limitations. First, a comprehensive history, which should include the patient’s lifestyle habits, medication history, allergies, family history, past medical conditions, and history of prior infections, was not obtained due to the patient’s inability to provide information at presentation and subsequent death. This significantly limits the assessment of potential contributing factors to the condition of the patient. Second, the lack of dermatological evaluation by the consultant dermatologist precludes a definitive diagnosis of SJS, and therefore, the diagnosis remains a probable diagnosis based on available findings. Due to lack of definitive information for the calculation of Algorithm of Drug Causality for Epidermal Necrolysis score and with complete precision, we were not able to calculate it and evaluate the likelihood of a drug-induced etiology. As such, while the temporal association with sofosbuvir and daclatasvir co-therapy raises suspicion, the causal relationship must be interpreted with caution. Despite these limitations, we believe it is crucial to report this case due to the rarity of the case in such a presentation. Reporting this case may raise clinical awareness and prompt further research. Future research and additional case reports will be essential to strengthen the evidence base and clarifying the potential association, thereby enhancing patient safety and care.

## Future directions

The benefits of sofosbuvir–daclatasvir combination regime to humans cannot be ignored, as it is considered to be the safest and most effective drug combination in modern medicine in HCV treatment plans. One rare but significant side effect of sofosbuvir–daclatasvir could be the development of SJS, which should not also be ignored by the physician, and patients should be educated that if any symptoms of SJS, like a rash, appear, they should promptly consult the doctor. Health care providers should remain vigilant and educate patients about the signs and symptoms of such adverse reactions. By ensuring timely intervention, the benefits of the sofosbuvir–daclatasvir combination can be maximized while minimizing potential risks.

## Conclusion

Most probably the case of SJS, an uncommon but potentially fatal mucocutaneous response due to sofosbuvir–daclatasvir therapy for hepatitis C virus (HCV) infection has been observed on the clinical practice. For patients using sofosbuvir–daclatasvir, it is critical to identify probable cases of SJS as soon as possible, stop the offending medication and begin supportive care. For Hep C, sofosbuvir–daclatasvir is a successful treatment, and their benefit to the humankind cannot be ignored; nevertheless, physicians should be aware of the possibility of SJS. In particular for patients who are at high risk, we advise routine skin examinations and educating the patient about this rare probable side effect and reporting of any rashes as soon as possible. It is essential to inform patients about the symptoms of SJS, which include fever, rash, and blisters. Clinical practices can lower hazards and improve patient safety by implementing these measures. Further investigation and monitoring are required to improve understanding of the risk factors and management approaches associated with SJS.

## Data Availability

Not applicable.
